# *Cortex phellodendri* Extract Relaxes Airway Smooth Muscle

**DOI:** 10.1155/2016/8703239

**Published:** 2016-04-27

**Authors:** Qiu-Ju Jiang, Weiwei Chen, Hong Dan, Li Tan, He Zhu, Guangzhong Yang, Jinhua Shen, Yong-Bo Peng, Ping Zhao, Lu Xue, Meng-Fei Yu, Liqun Ma, Xiao-Tang Si, Zhuo Wang, Jiapei Dai, Gangjian Qin, Chunbin Zou, Qing-Hua Liu

**Affiliations:** ^1^Institute for Medical Biology & Hubei Provincial Key Laboratory for Protection and Application of Special Plants in Wuling Area of China, College of Life Sciences, South-Central University for Nationalities, Wuhan 430074, China; ^2^Key Laboratory of Chinese Medicine Resource and Compound Prescription, Hubei University of Chinese Medicine, Wuhan 430065, China; ^3^College of Pharmacy, South-Central University for Nationalities, Wuhan 430074, China; ^4^Wuhan Institute for Neuroscience and Engineering, South-Central University for Nationalities, Wuhan 430074, China; ^5^Department of Medicine-Cardiology, Feinberg Cardiovascular Research Institute, Northwestern University Feinberg School of Medicine, Chicago, IL 60611, USA; ^6^Acute Lung Injury Center of Excellence, Division of Pulmonary, Allergy, and Critical Care Medicine, Department of Medicine, University of Pittsburgh School of Medicine, Pittsburgh, PA 15213, USA

## Abstract

*Cortex phellodendri* is used to reduce fever and remove dampness and toxin. Berberine is an active ingredient of* C. phellodendri*. Berberine from* Argemone ochroleuca* can relax airway smooth muscle (ASM); however, whether the nonberberine component of* C. phellodendri* has similar relaxant action was unclear. An n-butyl alcohol extract of* C. phellodendri* (NBAECP, nonberberine component) was prepared, which completely inhibits high K^+^- and acetylcholine- (ACH-) induced precontraction of airway smooth muscle in tracheal rings and lung slices from control and asthmatic mice, respectively. The contraction induced by high K^+^ was also blocked by nifedipine, a selective blocker of L-type Ca^2+^ channels. The ACH-induced contraction was partially inhibited by nifedipine and pyrazole 3, an inhibitor of TRPC3 and STIM/Orai channels. Taken together, our data demonstrate that NBAECP can relax ASM by inhibiting L-type Ca^2+^ channels and TRPC3 and/or STIM/Orai channels, suggesting that NBAECP could be developed to a new drug for relieving bronchospasm.

## 1. Introduction

Asthma is a common chronic respiratory disease [1]. Excessive airway obstruction is a cardinal symptom that results from the contraction of airway smooth muscle (ASM). In this study, we attempted to develop an effective and safe drug from bitter Chinese herbs to inhibit ASM contraction.

*Cortex phellodendri*, called Huang Bai in Chinese, which tasted bitter, is the dried bark of* Phellodendron chinense* Schneid. or* Phellodendron amurense* Rupr., which belongs to the group of Rutaceae arbor plants. It is bitter in flavor and cold in nature, categorized in kidney, urinary bladder, and large intestine meridians. The traditional functions are to clear heat, dry dampness, purge fire, and remove toxicity. It is one of fundamental traditional Chinese medicines. Previous study reported that* C. phellodendri* has many physiological activities, including antioxidant [2, 3], anti-inflammatory [4–6], antiulcer [7], and immune-stimulating properties [8], as well as neuroprotection and inhibition of coronavirus replication [9, 10]. Moreover,* C. phellodendri* combining with other herbs can reduce complications of corticosteroid-resistant asthma [11]. Berberine is one active ingredient of* C. phellodendri*; berberine from* Argemone ochroleuca* was demonstrated to have a relaxant effect in guinea-pig ASM [12]. However, whether the nonberberine component has similar relaxant action has not been investigated.

In the present study, we found that an n-butyl alcohol extract of* C. phellodendri* (NBAECP, nonberberine component) exerted inhibitory action on ASM contraction, and the underlying mechanism was also investigated.

## 2. Materials and Methods

### 2.1. *C. phellodendri* Extraction

*C. phellodendri*, bark of* Phellodendron chinensis* Schneid. (Rutaceae), were collected in Sichuan Province, China, and were authenticated by Professor Dr. Ding-Rong Wan of our university. A voucher specimen (SCUN201310010) is deposited at the Herbarium of College of Pharmacy, South-Central University for Nationalities, China.

Air-dried* C. phellodendri* (1 Kg) was milled into powder and immersed into 70% ethanol (5 L) for 24 h. The components in the mixture were extracted by hot reflux and were centrifuged. The supernatant was collected and evaporated to dryness under reduced pressure using a rotary evaporator to remove ethanol and get residues, which were immersed in a 2% HCl solution (1000 mL). The yellow precipitates (mainly berberine) were removed and the supernatants were consecutively extracted with petroleum ether, chloroform, ethyl acetate, and n-butyl alcohol. The n-butyl alcohol extract was further evaporated under reduced pressure, and the extraction yield was 1.5% of the raw material dry weight. The dried n-butyl alcohol extract of* C. phellodendri* (NBAECP) was dissolved in 3% DMSO for the experiments.

### 2.2. Reagents

Nifedipine, acetylcholine chloride (ACH), and pyrazole 3 (Pyr 3) were purchased from Sigma Chemical Co. (St. Louis, MO, USA); DMEM was purchased from Gibco BRL Co. (Invitrogen Life Technologies, Carlsbad, CA, USA). Other chemicals were purchased from Sinopharm Chemical Reagent Co. (Shanghai, China).

### 2.3. Animals

Sexually mature male BALB/c mice were purchased from the Hubei Provincial Center for Disease Control and Prevention (Wuhan, China). The mice were housed at room temperature (20–25°C) and constant humidity (50–60%) under a 12 h light-dark cycle in an SPF grade animal facility. The experiments on animals were approved by the Animal Care and Ethics Committee of the South-Central University for Nationalities and conformed to the guidelines of the Institutional Animal Care and Use Committee of the South-Central University for Nationalities (QHL-6, 12-10-2013).

### 2.4. Experimental Asthma Model in Mice

Asthmatic mice were prepared as described previously [13]. Briefly, mice were sensitized by intraperitoneal injection administration of 0.2 mL of 0.9% saline solution containing 0.6 mg OVA and 0.4 mg of adjuvant aluminum hydroxide on days 1 and 8; then, the mice were challenged from days 15 through 19 by daily intranasal instillation of 50 *μ*L of OVA solution (3 mg/mL). Control mice were sensitized and challenged by identical vehicle media.

### 2.5. Tracheal ASM Contraction Measurement

Mouse ASM contraction was measured as previously described [14]. Adult male BALB/c mice were sacrificed by an intraperitoneal injection of sodium pentobarbital (150 mg/kg), and their tracheae were isolated and quickly transferred to ice cold PSS (composition in mM: NaCl 135, KCl 5, MgCl_2_ 1, CaCl_2_ 2, HEPES 10, glucose 10, pH 7.4). The connective tissue was removed, and tracheal rings (~5 mm) were cut from the bottom of the tracheae. Each ring was mounted with a preload of 0.5 g in an organ bath with a 10 mL capacity containing PSS bubbled with 95% O_2_ and 5% CO_2_ at 37°C. The rings were equilibrated for 60 min, precontracted with high K^+^ (80 mM) or ACH (10^−4^ M), washed, and rested for a total of 3 times. The experiments were performed following an additional 30 min rest.

### 2.6. Bronchial ASM Contraction Measurement

Lung slices were prepared according to a previous report [15]. Lung slices were placed in a chamber and were held with a small nylon mesh. Perfusion was maintained in Hanks' balanced salt solution (HBSS) at a rate of ~800 *μ*L/min. HBSS was supplemented with 20 mM HEPES buffer (composition in mM: NaCl 137.93, KCl 5.33, NaHCO_3_ 4.17, CaCl_2_ 1.26, MgCl_2_ 0.493, MgSO_4_ 0.407, KH_2_PO 0.4414, Na_2_HPO4 0.338, and D-glucose 5.56) and adjusted to a pH of 7.4. Images of lung slices under 10x objective were acquired at the rate of 30 frames/min using an LSM 700 laser confocal microscope (Carl Zeiss, Goettingen, Germany). The cross-sectional area of the bronchial lumen was measured using Zen 2010 software (Carl Zeiss, Goettingen, Germany). The experiments were performed at room temperature.

### 2.7. Data Analysis

The results are expressed as the mean ± SEM. Comparisons of 2 groups were performed using Student's* t*-test. Differences with *P* < 0.05 were considered significant.

## 3. Results

### 3.1. NBAECP Inhibits High K^+^-Induced Tracheal Smooth Muscle Contraction

**Figure 1:**
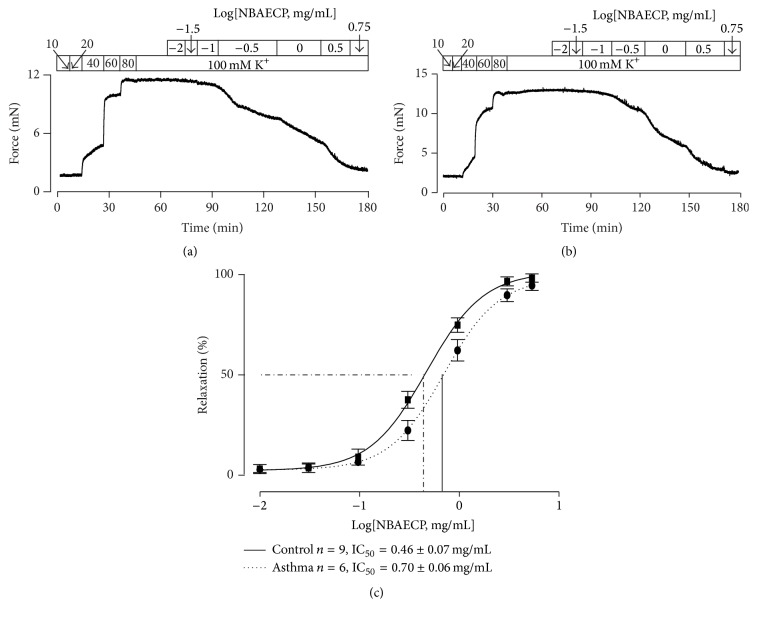
NBAECP inhibits high K^+^-induced contraction in TRs. (a) High K^+^ triggered contractions in a healthy (i.e., control) TR, which reached the maximum at 80 mM K^+^. Following cumulative additions of NBAECP, the sustained contraction was totally blocked. (b) An identical experiment was performed using an asthmatic TR, and a similar result was observed. (c) Dose-relaxation relationships of NBAECP from 9 control and 6 asthmatic TRs. The IC_50_ of NBAECP was 48.9 ± 1.5 *μ*g/mL (*n* = 9) in control TRs and 73.1 ± 1.6 *μ*g/mL (*n* = 6) in asthmatic TRs. These data demonstrated that NBAECP could block high K^+^-induced precontraction in control and asthmatic tracheal smooth muscle.

To observe the effect of NBAECP on the contraction of airway smooth muscle (ASM), the tracheal rings (TRs) from healthy mice (i.e., controls) were contracted using high K^+^. Following the increase in the K^+^ concentration from 10 to 80 mM, the TRs exhibited dose-dependent contraction (Figure 1(a)). Upon the contraction reaching the maximum (at 80 mM K^+^), NBAECP was added. The contraction was inhibited in a dose-dependent manner. An identical experiment was also performed in a TR from an asthmatic model mouse, and similar results were observed (Figure 1(b)). NBAECP-induced relaxation in both control and asthmatic TRs was analyzed, and the values of half maximal inhibitory concentration (IC_50_) were calculated (Figure 1(c)). There were no significant differences between the two traces and the values of IC_50_. These results indicated that NBAECP could inhibit agonist-induced sustained contraction of control and asthmatic ASM.

### 3.2. NBAECP Blocks ACH-Induced Tracheal Smooth Muscle Contraction

**Figure 2:**
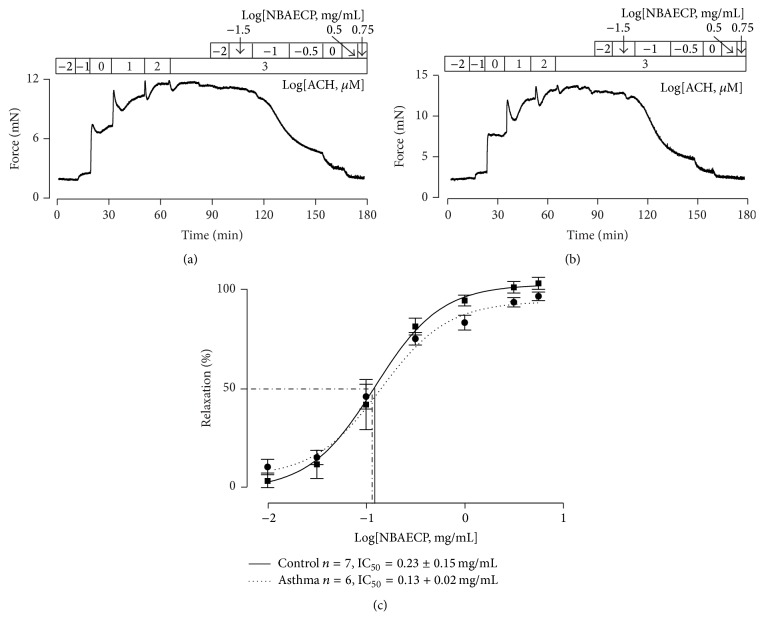
NBAECP inhibits ACH-induced precontraction in TRs. (a) Following cumulative addition of ACH, a TR reached a sustained contraction, which was inhibited following cumulative application of NBAECP. (b) A similar experiment was performed in asthmatic TR. (c) The summary results of NBAECP-induced relaxation in 7 control and 6 asthmatic TRs. The IC_50_ of NBAECP was 12.2 ± 1.3 *μ*g/mL in control TRs and 12.8 ± 1.2 *μ*g/mL in asthmatic TRs. These results indicated that NBAECP could block ACH-induced sustained contractions in control and asthmatic tracheal smooth muscle.

To know whether NBAECP is capable of inhibiting another agonist-induced precontraction in ASM, healthy TRs were contracted using ACH. Upon the contraction reaching the maximum, NBAECP was added (Figure 2(a)). Similar dose-dependent relaxation responses occurred. Moreover, these responses existed in asthmatic TRs (Figure 2(b)). The dose-relaxation relationships and IC_50_ values of NBAECP were analyzed (Figure 2(c)), and they did not show differences between the control and asthmatic TRs. These experiments demonstrated that NBAECP could also inhibit ACH-induced precontraction in control and asthmatic ASM.

**Figure 3:**
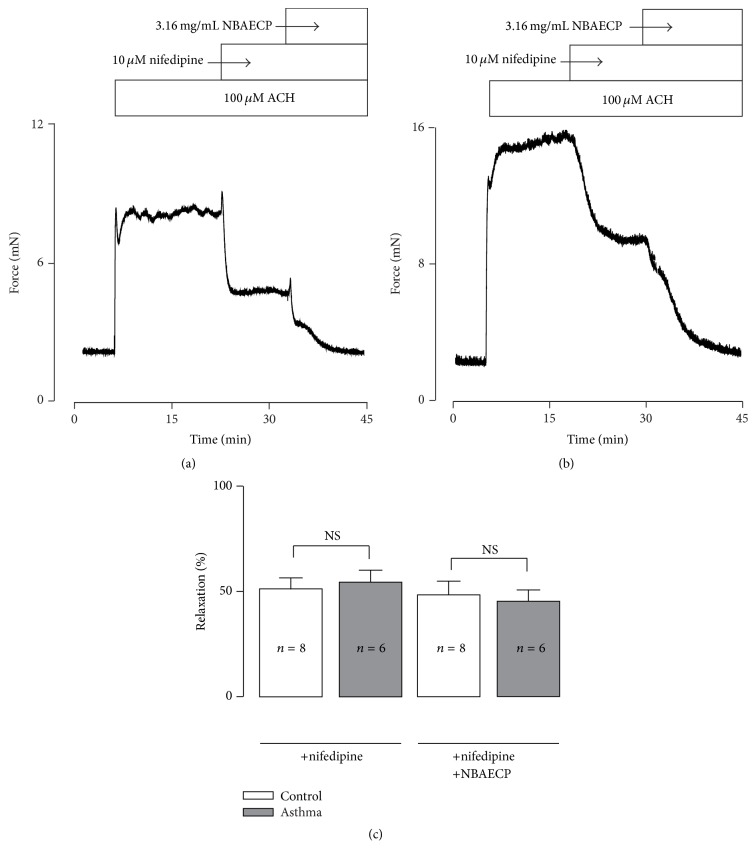
Nifedipine partially inhibits ACH-caused contraction. (a) ACH (100 *μ*M) induced a sustained contraction in a control TR, which was partially inhibited by nifedipine (10 *μ*M). The remaining contract was further blocked by NBAECP (3.16 mg/mL). (b) An identical experiment was performed in an asthmatic TR. (c) The summary data from 8 control and 6 asthmatic TRs. ^NS^ *P* > 0.05. These data demonstrated that activation of L-type Ca^2+^ channels played a role in ACH-induced contraction, and NBAECP could inhibit nifedipine-resistant channels, resulting in total relaxation.

Were these relaxation responses also mediated by L-type Ca^2+^ channels? To answer this question, TRs from healthy and asthmatic mice were precontracted with ACH, and 10 *μ*M nifedipine, a selective blocker of voltage-dependent L-type Ca^2+^ channels (VDCCs), was then added (Figures 3(a) and 3(b)). Following the addition of nifedipine, the contractions were partially inhibited. The resistant components were further blocked by NBAECP. The inhibitions induced by nifedipine and NBAECP were not different between the control and asthmatic TRs (Figure 3(c)).

**Figure 4:**
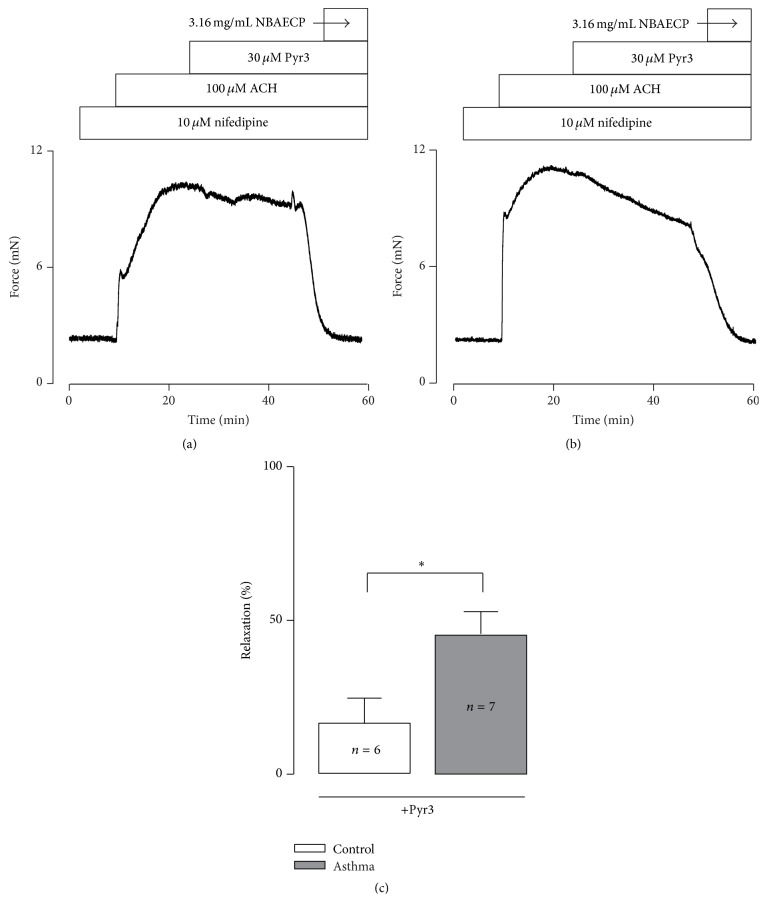
Pyr 3 partially inhibits ACH-induced contraction. (a) In the presence of nifedipine (10 *μ*M), ACH-induced sustained contraction was partially inhibited by Pyr 3 (a blocker of TRPC3 and STIM/Orai channels) and then was completely blocked by NBAECP. (b) The same experiment was performed in an asthmatic TR. (c) Summary results from 6 control and 7 asthmatic TRs. ^*∗*^ *P* < 0.05. These results indicated that activation of TRPC3 and/or STIM/Orai channels participated in ACH-induced contraction, and these channels were inhibited by NBAECP, thus resulting in relaxation.

To further investigate the underlying mechanism of NBAECP-induced relaxation of the nifedipine-resistant component, TRs were incubated with 10 *μ*M nifedipine for 10 min and contracted using ACH, and we then observed the action of Pyr 3 (an inhibitor of TRPC3 and STIM/Orai channels). The results showed that Pyr 3 induced partial relaxation, and the remaining contractions were completely blocked by NBAECP (Figures 4(a) and 4(b)); however, Pyr 3-induced relaxation in asthmatic TRs was markedly greater than in the controls (Figure 4(c)).

Taken together, these results indicated that NBAECP-induced relaxation responses were mediated by L-type Ca^2+^, TRPC3, and/or STIM/Orai channels.

### 3.3. NBAECP Inhibits Bronchial Smooth Muscle Contraction

**Figure 5:**
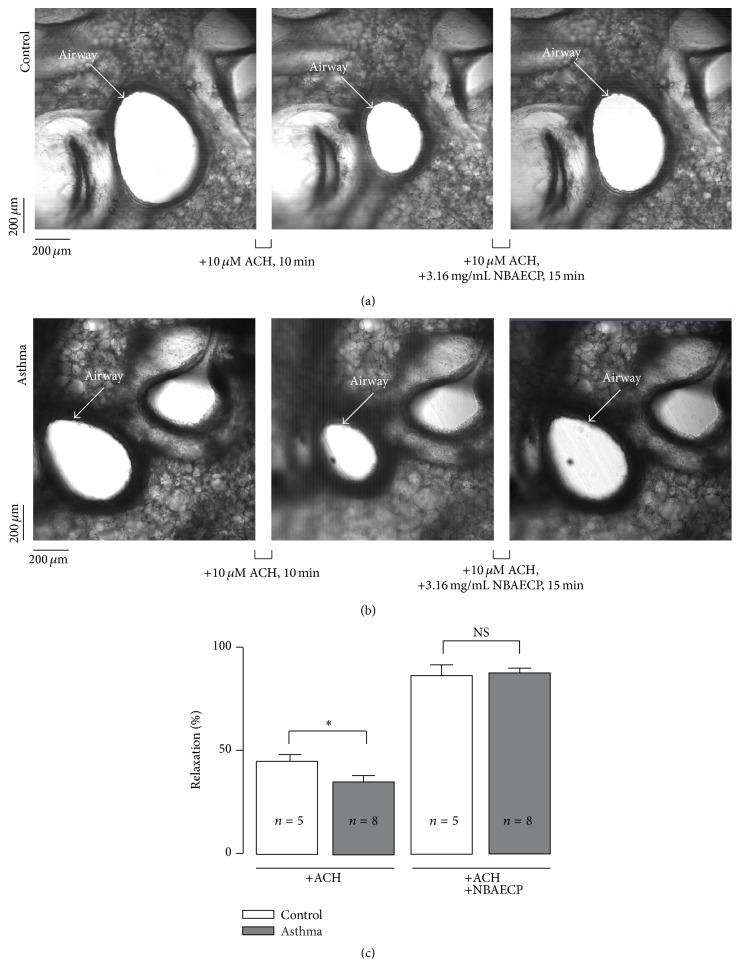
NBAECP inhibits bronchial smooth muscle contraction. (a) The intrapulmonary airway in a lung slice from a control mouse (left) following addition of ACH (10 *μ*M for 10 min): the cross-section area of the airway lumen decreased (middle); then upon application of NBAECP (3.16 mg/mL for 15 min), the cross-section area of the airway lumen increased (right). (b) Similar measurements in an asthmatic lung slice. (c) Summary data from 8 slices/8 control mice and 5 slices/5 asthmatic mice. ^*∗*^ *P* < 0.05, ^NS^ *P* > 0.05. These experiments demonstrated that NBAECP could inhibit ACH-induced contraction in bronchial smooth muscle.

To define whether NBAECP has similar relaxant action on bronchial smooth muscle, lung slices were cut, and the cross-sectional area of the airway lumen was measured. The area of the airway lumen from healthy mice markedly decreased following application of 100 *μ*M ACH; however, it was restored upon addition of 3.16 mg/mL NBAECP (Figure 5(a)). Identical experiments were conducted in lung slices from asthmatic mice, and similar phenomena were observed (Figure 5(b)). The summary data are shown in Figure 5(c). These results indicated that NBAECP had similar relaxant action on bronchial smooth muscle.

## 4. Discussion

In the present study, our data demonstrate that NBAECP can inhibit L-type Ca^2+^ channels, blocking high K^+^-induced contractions in healthy and asthmatic airway smooth muscle and additionally inhibiting TRPC3 and/or STIM/Orai channels to reduce ACH-induced contractions in both types of airway smooth muscle. These results indicate that NBAECP could be a new bronchodilator for the treatment of asthma.

The purpose of this study was to find bronchodilators among Chinese medicines. We extracted a component (NBAECP, nonberberine active ingredient) from* C. phellodendri*. To investigate whether NBAECP has relaxant action, we used high K^+^ and ACH to contract airway smooth muscle and then observed the effect of NBAECP. NBAECP totally relaxed high K^+^-induced precontractions (Figure 1). High K^+^ could induce membrane depolarization, resulting in the activation of voltage-dependent L-type Ca^2+^ channels (VDCCs) [16]. The channels then mediated Ca^2+^ influx, resulting in intracellular Ca^2+^ concentration increases, leading to muscle contraction. Nifedipine, a selective blocker of VDCCs, completely blocked high K^+^-induced contractions. This phenomenon was observed in our previous study [14, 17]. These results indicated that high K^+^-induced contractions completely depended on L-type Ca^2+^ channel-mediated Ca^2+^ influx. Thus, NBAECP-induced complete inhibition of high K^+^-induced contraction due to NBAECP resulted in the inhibition of L-type Ca^2+^ channels, thus terminating Ca^2+^ influx. However, the inhibitory mechanism of NBAECP on L-type Ca^2+^ channels must be further investigated.

Airway smooth muscle expresses the muscarinic (M) receptor family, which includes 5 subtypes (M1–M5) [18]. Among them, G protein-coupled M3 plays a more important role in the contraction of airway smooth muscle [19]. Stimulation of M3 by agonists results in intracellular Ca^2+^ release from the sarcoplasmic reticulum (SR) via the PLC-IP3-IP3R pathway [20]. This leads to intracellular Ca^2+^ concentrations increasing and further triggering airway smooth muscle contraction. However, this pathway only mediates transient contractions [21], while the sustained contraction depends on Ca^2+^ influx from the extracellular side and/or Ca^2+^ sensitization [22, 23]. Hence, the M3 agonist ACH-induced sustained contraction in airway smooth muscle (Figure 2) was probably due to Ca^2+^ influx. Previous reports have demonstrated that L-type Ca^2+^ channels play roles in ACH-induced airway smooth muscle contraction [24]. These findings were further confirmed in this study, in which nifedipine partially inhibited ACH-induced contraction in TRs (Figure 3).

Moreover, TRPC3 and/or STIM/Orai channels also play roles in ACH-induced contractions [14, 25]. TRPC3 and STIM/Orai channels are nonselective cation channels (NSCCs) that can mediate Ca^2+^ influx, resulting in intracellular Ca^2+^ increase to trigger airway smooth muscle contraction and contributing to ACH-induced contractions [26]. In our data, following the addition of Pyr 3 (blocker of TRPC3 and STIM/Orai channels), ACH-induced contractions were partly inhibited (Figure 3). In addition to L-type Ca^2+^ channels and two types of NSCCs, there are still other mechanisms that mediate ACH-induced contraction on the basis of nifedipine-resistant and Pyr 3-resistant contractions being further blocked by NBAECP (Figure 4). These unknown mechanisms must be further determined in the future.

Taken together, the above results indicate that ACH-induced sustained contraction results from L-type Ca^2+^ channels- and TRPC3 and/or STIM/Orai channels-mediated Ca^2+^ influx and unknown mechanisms. Thus, NBAECP-induced inhibition could be partially due to NBAECP inhibiting these channels. However, the detailed inhibitory mechanism requires further investigation.

Although the above data showed that NBAECP could inhibit agonist-induced precontractions in tracheal smooth muscle, whether it has similar inhibitory functions on bronchial smooth muscle is uncertain. Our experiments conducted in lung slices showed that NBAECP was also able to inhibit precontraction in small bronchial smooth muscle (Figure 5), indicating that NBAECP could inhibit whole airway smooth muscle contraction.

In addition, in this study, all of the experiments were performed in both healthy and asthmatic smooth muscles, and similar responses were observed. This finding indicates that NBAECP has similar inhibitory roles in both types of ASM, suggesting that NBAECP could be a potent bronchodilator for asthmatics.

## 5. Conclusions

NBAECP can inhibit agonist-induced sustained contractions of healthy and asthmatic airway smooth muscle by inhibiting several types of ion channels. These findings indicate that NBAECP could be a new inhibitor of asthma attacks.
